# Identification of central genes for endometriosis through integration of single-cell RNA sequencing and bulk RNA sequencing analysis

**DOI:** 10.1097/MD.0000000000036707

**Published:** 2023-12-15

**Authors:** Yulin Song, Le Wang, Yu Zhang

**Affiliations:** a Department of obstetrics and gynecology, Qinhuangdao Maternal and Child Health Hospital, Qinhuangdao, Hebei, China; b Department of Neurology, Shaanxi Provincial People’s Hospital, Xi’an, Shaanxi, China; c Department of Gynecology, Shaanxi Provincial People’s Hospital, Xi’an, Shaanxi, 710068, China.

**Keywords:** biomarkers, endometriosis, inflammation, single-cell RNA sequencing

## Abstract

This study aimed to identify the key genes involved in the development of endometriosis and construct an accurate predictive model to provide new directions for the diagnosis and treatment of endometriosis. Using bioinformatics analysis, we employed the single-cell cell communication method to identify the key cell subtypes. By combining chip data and integrating differential analysis, WGCNA analysis, and the least absolute shrinkage and selection operator (LASSO) model, key genes were identified for immune infiltration and functional enrichment analyses. Cell communication analysis identified tissue stem cells as the key subtype. Differential analysis revealed 1879 differentially expressed genes, whereas WGCNA identified 357 module genes. The LASSO model further selects 4 key genes: Adipocyte Enhancer Binding Protein 1(AEBP1), MBNL1, GREM1, and DES. All 4 key genes showed significant correlations with immune cell content. Moreover, these genes were significantly expressed in single cells. The predictive model demonstrated good diagnostic performance. Through scRNA-seq, WGCNA, and LASSO methodologies, DES, GREM1, MBNL1, and AEBP1 emerged as crucial core genes linked to tissue stem cell markers in endometriosis. These genes have promising applications as diagnostic markers and therapeutic targets for endometriosis.

## 1. Introduction

Endometriosis is a gynecological disorder characterized by endometrial-like tissue appearing beyond the confines of the uterus, and is driven by estrogen dependency.^[1]^ It impacts approximately 190 million women worldwide, accounting for approximately 10% of all women of childbearing age.^[2]^ The growth of endometrial tissue outside the uterus results in lesions that cause chronic pelvic pain, irregular menstrual bleeding, and infertility.^[3,4]^ Due to a range of reasons, there is an average delay of 7 years between the emergence of symptoms and the surgical diagnosis of endometriosis.^[5]^ Although the pathogenesis of endometriosis involves retrograde menstruation, hormones, immunologic determinants, stem cells, genetic/epigenetic factors, coelomic metaplasia, Müllerian duct remnants, and lymphatic and vascular dissemination,^[6,7]^ a comprehensive understanding of this disease development is still lacking. The prevalence of endometriosis and delayed diagnosis significantly impact the overall well-being of women, both physically and mentally. Moreover, they contribute to increased healthcare costs at the individual and national levels, while mounting economic pressure. Consequently, there is a pressing need to investigate its pathogenesis further, explore innovative diagnostic methods, and identify novel therapeutic targets.

The regurgitation of endometrial tissue fragments/cells and protein-rich fluid from the uterus through the fallopian tubes into the pelvic cavity during menstruation is commonly regarded as the primary contributor to the development of endometriotic lesions within the peritoneal cavity. Nonetheless, this mechanism alone cannot fully account for their occurrence as retrograde menstruation is a common experience in nearly all women.^[8,9]^ This prompts us to contemplate whether particular cells within the endometrial tissue during menstruation hold a crucial role in the formation of endometriosis. Single-cell transcriptome sequencing, also called scRNA-seq, was used to measure gene expression at the cellular level. This allows for a higher resolution for distinguishing cell types compared to traditional bulk RNA-seq.^[10]^ Through the utilization of scRNA-seq, researchers have made significant strides in identifying new cell types,^[11]^ comprehending the dynamics of developmental processes at a granular level,^[12]^ and unraveling intricate gene regulatory mechanisms.^[13]^ Furthermore, extensive investigations have been conducted to explore the use of single-cell sequencing for the study of endometriosis. Through single-cell RNA sequencing, we uncovered the complex and diverse nature of fibrosis and angiogenesis associated with endometriosis, shedding light on its inherent heterogeneity.^[14]^ Additionally, single-cell RNA sequencing has yielded crucial findings regarding the existence of specific cell subtypes and immune dysfunction in the peritoneal fluid of individuals diagnosed with endometriosis.^[15]^ By harnessing the power of scRNA-seq, we can obtain intricate and precise data, leading to significant advancements in our understanding of cellular function, disease progression, and treatment effectiveness.^[16]^ Hence, this study aimed to investigate the essential genes involved in endometriosis by integrating single-cell sequencing data with transcriptome analysis.

Using bioinformatics analysis, in conjunction with single-cell RNA sequencing (scRNA-seq) and a vast amount of bulk sequencing data, we successfully identified the crucial genes involved in endometriosis. Furthermore, we developed a diagnostic model to identify novel diagnostic approaches to endometriosis. The analysis process can be seen in supplementary Figure 1, http://links.lww.com/MD/L111. This study contributes to experimental research and establishes a theoretical framework for the development of therapeutic drugs.

## 2. Methods

### 2.1. Data acquisition

The single-cell dataset GSE203191 was obtained by downloading the data file from the National Center for Biotechnology Information (NCBI) Gene Expression Omnibus (GEO) public database, which comprises complete expression profiles from 11 cases. The Series Matrix File data of GSE25628 (GPL571) were also acquired, consisting of expression profile data from 13 patients, including 6 in the normal group and 7 in the disease group. Similarly, the Series Matrix File data of GSE11691 (GPL96) were downloaded, containing expression profile data from 18 patients, with 9 in the normal group and 9 in the disease group. Additionally, the Series Matrix File data of GSE7305 (GPL570) were obtained, encompassing the expression profile data from 20 patients divided into 10 cases in the normal group and 10 cases in the disease group. The GSE11691 and GSE7305 datasets were merged and batch correction between chips was performed using the combat algorithm. The data used in this study is publicly available from the GEO database, which collects studies where ethical approval has been granted by the respective institutions responsible for the original studies. Therefore, additional ethical approval for our study was not required and no further patient consent was needed as the data is anonymized and de-identified.

### 2.2. Single cell analysis

Initially, the expression profiles were read in the Seurat package, followed by the removal of low-expression genes (nFeature RNA > 50 and mt < 5). Subsequently, the data were subjected to standardization, normalization, and principal component analysis (PCA). Optimal PC numbers were observed using ElbowPlot, whereas t-distributed Stochastic Neighbor Embedding (t-SNE) analysis revealed positional relationships between clusters. Cluster annotation was performed using the Celldex package, and cells that were important for disease occurrence were annotated. Finally, the logfc.threshold parameter was set to 1 in FindAllMarkers to extract marker genes for each cell subtype from the single-cell expression profiles. Genes meeting the criteria of |avg log2FC| > 1 and p val adj < 0.05 were considered as unique marker genes within each cell subtype.

### 2.3. Ligand receptor interaction analysis (Cellchat)

CellChat is a powerful tool that allows the quantitative inference and analysis of intercellular communication networks using single-cell data. By employing network analysis and pattern recognition techniques, CellChat predicted the primary signal inputs and outputs of cells and elucidated their functional coordination. In this study, we utilized normalized single-cell expression profiles as input data, whereas cell subtypes derived from single-cell analysis served as cell information. We examined cell-related interactions by quantifying the proximity of these relationships using interaction strength (weight) and frequency (count). This approach enabled observation of the activity and influence of each cell type on the disease.

### 2.4. Differential expression analysis

The Limma package was used for precise differential expression analysis of the expression profiles. The purpose of this study was to identify differentially expressed genes (DEGs) between the groups. In this study, the “limma” R package was used to analyze the molecular mechanisms within the data and detect differentially expressed genes between control and disease samples. The screening criteria for differential gene analysis were set at |logFC| > 0.585 and *P* < .05. Additionally, a volcano plot was generated to visualize the differential gene expression.

### 2.5. Weighted Gene Co-expression Network Analysis (WGCNA)

By constructing a weighted gene co-expression network, we aimed to uncover co-expressed gene modules, investigate their relevance to disease, and identify key genes within the network. The WGCNA-R package served as a vital tool for constructing a gene co-expression network using our dataset. Subsequently, using this algorithm, we screened the top 5000 genes with the highest variance to facilitate further analysis. To estimate network connectivity, we converted the weighted adjacency matrix into a topological overlap matrix (TOM). Employing hierarchical clustering, we constructed a clustering tree structure based on the TOM matrix, with different branches representing distinct gene modules, each assigned a unique color. By employing the weighted correlation coefficient, genes were classified based on their expression patterns and grouped accordingly, leading to the division of tens of thousands of genes into multiple modules based on their expression patterns.

### 2.6. Prediction model construction

Following the selection of module genes, a prediction model was developed using Least Absolute Shrinkage and Selection Operator (LASSO) regression. Through integration of gene expression values, a risk score formula was created for each patient. This formula was then weighted using the estimated regression coefficients obtained from LASSO regression analysis. Subsequently, patient scores were computed based on the risk score formula, and the accuracy of the model predictions was evaluated using the Receiver Operating Characteristic (ROC) curve.

### 2.7. Immune infiltration assay

We employed the CIBERSORT algorithm to analyze RNA-seq data obtained from diverse patient subgroups, enabling us to infer the relative proportions of immune-infiltrating cells. Through this analysis, we explored the interactions between the immune cells and assessed their impact. To evaluate the effect of the genes on immune cell infiltration, we visualized the relative contents of the immune cells. Spearman correlation analysis was performed to examine the association between gene expression and immune cell content. Statistical significance was set at a threshold of *P* < .05.

### 2.8. Gene set variation analysis (GSVA)

GSVA is a nonparametric and unsupervised approach that enables evaluation of gene set enrichment in the transcriptome. By systematically scoring the gene set of interest, GSVA transforms gene-level changes into pathway-level changes, thereby facilitating the assessment of the biological function of the sample. Gene sets were obtained from a molecular signature database. Using the GSVA algorithm, we comprehensively scored each gene set to assess potential changes in biological function across different samples.

### 2.9. Gene Set Enrichment Analysis (GSEA)

Patients were stratified into high- and low-expression groups based on the expression levels of key genes. Subsequently, GSEA was used to analyze disparities in signaling pathways between the 2 groups. The background gene set used for analysis was the annotated gene set obtained from the MsigDB database (version 7.0), which served as a reference for subtype pathway annotation. Differential expression analysis of pathways across different groups was conducted and significantly enriched gene sets were ranked based on their consistency scores (adjusted *P* < .05). GSEA is a valuable tool that is commonly employed to explore the intricate connection between disease classification and biological significance.

### 2.10. MicroRNA(miRNA) network construction

MicroRNAs (miRNAs) are a class of small non-coding RNAs that play significant roles in the regulation of gene expression by facilitating mRNA degradation or inhibiting mRNA translation. Therefore, we investigated whether certain miRNAs associated with key genes exert regulatory control over the transcription or degradation of hazardous genes. To identify miRNAs related to the key genes, we used the miRcode database. Using the Cytoscape software, we visualized the miRNA-gene network and gained valuable insights into their intricate interactions.

### 2.11. Regulatory network analysis of key genes

In this study, we employed the “RcisTarget” R package to predict transcription factors using motif-based calculations. Uniquely, the normalized enrichment score (NES) of a motif is influenced by the total number of motifs present in the database. To augment motif annotations, additional annotation files were generated based on motif similarity and gene sequence analysis. The initial step in assessing the overexpression of each motif across a given gene set involved calculating the area under curve (AUC) for every motif-motif-set combination. This computation relies on plotting the recovery curve of the gene set against the ordering of the motifs. Ultimately, the NES of each motif was derived from the AUC distribution encompassing all the motifs within the gene set.

### 2.12. Statistical analysis

In our study, we utilized R language (version 4.2.2) for all statistical analyses. Specifically, we employed various bioinformatics packages and algorithms such as Seurat for single-cell analysis, CellChat for analysis of intercellular communication networks, Limma for differential expression analysis, WGCNA for constructing a gene co-expression network, LASSO regression for prediction model construction, CIBERSORT for immune infiltration assay, GSVA and GSEA for enrichment analysis, and RcisTarget for predicting transcription factors. The significance level was set at *P* < .05 to determine statistical significance.

## 3. Results

### 3.1. Single-cell level analysis in scRNA-Seq data

**Figure 1.**
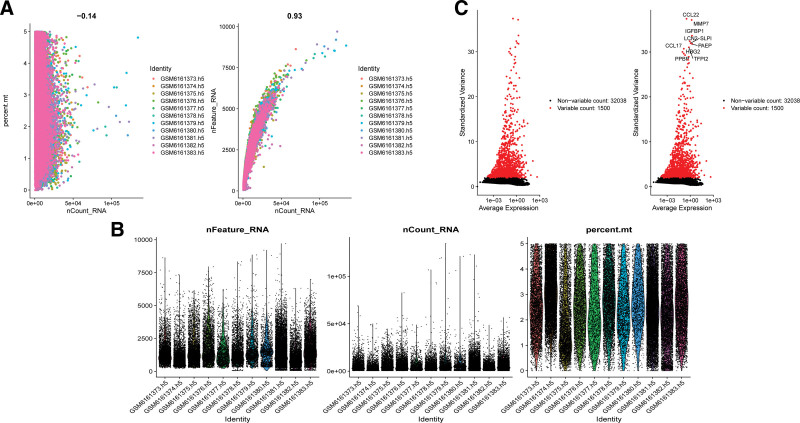
Characterization of endometriosis by Single-cell RNA sequencing (scRNA-seq). (A) The left figure shows the relationship between cell sequencing depth and mitochondrial content, and the right figure shows the relationship between sequencing depth and gene quantity, and the 2 are positively correlated. (B) Single-cell quality control showing cell number, gene number, and sequencing depth for each sample. (C) Genes that were significantly different between cells were identified and the characteristic variance was plotted.

**Figure 2.**
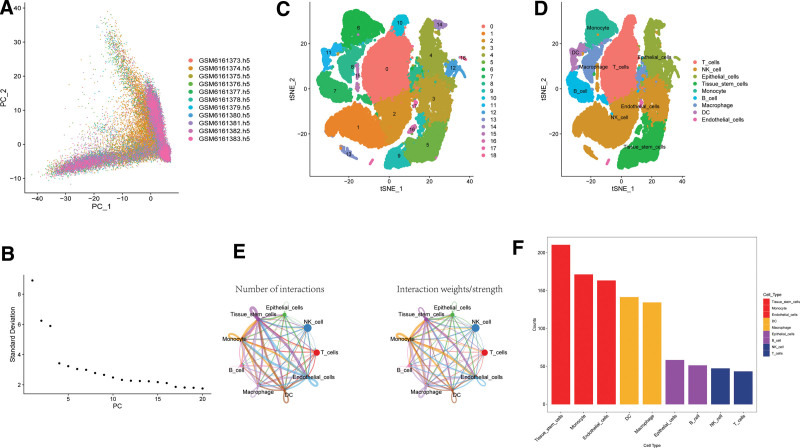
Cell annotation and cell communication analysis. (A) The display of principal component analysis (PCA) and the distribution of PCs, where dots represent cells and colors represent samples. (B) The variance ranking plot of each PC. (C) Using the important components available in PCA, the cells were divided into 19 clusters using t-Distributed Stochastic Neighbor Embedding (t-SNE) algorithm. (D) Cell annotation of 20 clusters. 20 clusters are annotated into 9 cell types, namely T_cells, NK_cell, Epithelial_cells, Tissue_stem_cells, Monocyte, B_cell, Macrophage, DC and Endothelial_cells. (E) The cell-cell interaction network among the 9 cell types, where the width of edges represents the probability and strength of communication between cells. (F) The comparison of total interactions in the communication network among the 9 cell types, decreasing from left to right, with Tissue_stem_cells being the strongest.

Single-cell GSE203191 data were obtained from the NCBI for Biotechnology Information GEO public database. To ensure data quality, samples were initially filtered based on nFeature RNA and nCount RNA (nFeature RNA > 50%. mt < 5) (Fig. 1A and B). The top 10 genes with the highest standard deviations are shown (Fig. 1C). PCA dimensionality reduction analysis revealed no significant batch effect between the samples (Fig. 2A), and the optimal number of principal components was determined using ElbowPlot10 (Fig. 2B). Ultimately, the TSNE analysis identified 19 distinct subgroups (Fig. 2C).

### 3.2. Annotation of cell subsets and cell communication of single cell data

In this study, we employed the R package SingleR to annotate each subtype, resulting in the annotation of 19 clusters into 9 distinct cell types: T cells, NK cells, epithelial cells, tissue stem cells, monocytes, B cells, macrophages, DC, and endothelial cells (Fig. 2D). To investigate ligand-receptor relationships within the single-cell expression profile, we utilized the Cellchat software package. Our analysis revealed intricate pairs of interactions among these cell subtypes (Fig. 2E). Notably, through statistical analysis, we found that tissue stem cells, monocytes, and other cell types exhibited close potential interactions with other cells (Fig. 2F). Consequently, we selected the marker genes of the tissue stem cells as the candidate gene set.

### 3.3. Differential gene analysis and WGCNA network construction

**Figure 3.**
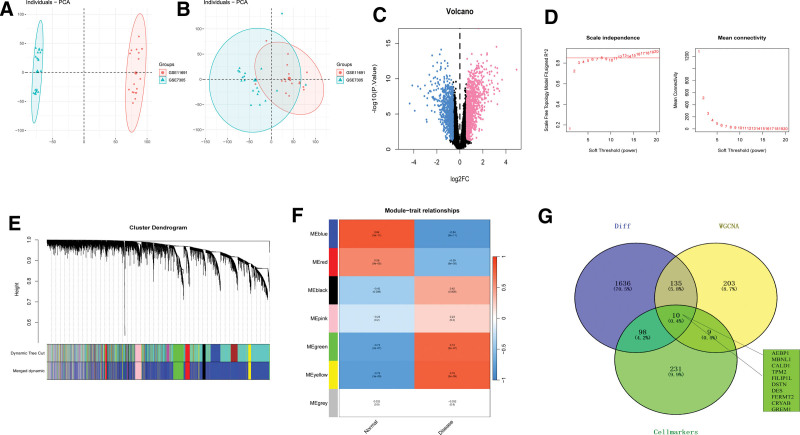
Screening for endometriosis signature genes. (A, B) The datasets GSE11691 and GSE7305 were corrected for batch effects using the combat algorithm. (C) The volcano plot of differentially expressed genes, with pink indicating upregulation and blue indicating downregulation. (D) The scale-free index and average connectivity of different soft thresholds in endometriosis. (E) The dendrogram of gene clustering in endometriosis illustrates different modules represented by distinct colors. (F) The heatmap demonstrates the correlation between module-specific genes and endometriosis, with blue indicating negative correlation and red indicating positive correlation. (G) The Venn diagram identifies the feature genes.

Datasets related to endometriosis, GSE11691 and GSE7305, were downloaded from the GEO database. The study included the expression profiles of 38 patients, comprising a control group (n = 19) and disease group (n = 19). To correct for the chip-based variation, we employed the SVA algorithm and visualized the batch effect using PCA. The results demonstrated a reduction in the batch effect between chips following the correction (Fig. 3A and B). Differential gene analysis was performed using the limma package to identify genes that exhibited significant differences in expression between the control and disease groups. The screening criteria for differential genes were set at |logFC|>0.585 and *P* < .05, resulting in the identification of 1879 differentially expressed genes (Fig. 3C). Among these, 1057 genes were upregulated, whereas 822 genes were downregulated. Furthermore, a WGCNA network was constructed to investigate the co-expression network of the genes in the endometriosis cohort. With the soft threshold β set to 8 (Fig. 3D), gene modules were detected based on the tom matrix. Seven gene modules were identified (Fig. 3E and F), namely black (177), blue (3036), green (349), gray (729), red (181), pink (171), and yellow (357). Notably, the yellow module exhibited the strongest positive correlation with disease (cor = 0.79, p = (3e − 09)), making it the focus of subsequent analyses. By intersecting 1879 differential genes, yellow module genes, and marker genes of tissue stem cells, a total of 10 overlapping genes were identified (Fig. 3G).

### 3.4. Construction of prediction model and identification of key genes

**Figure 4.**
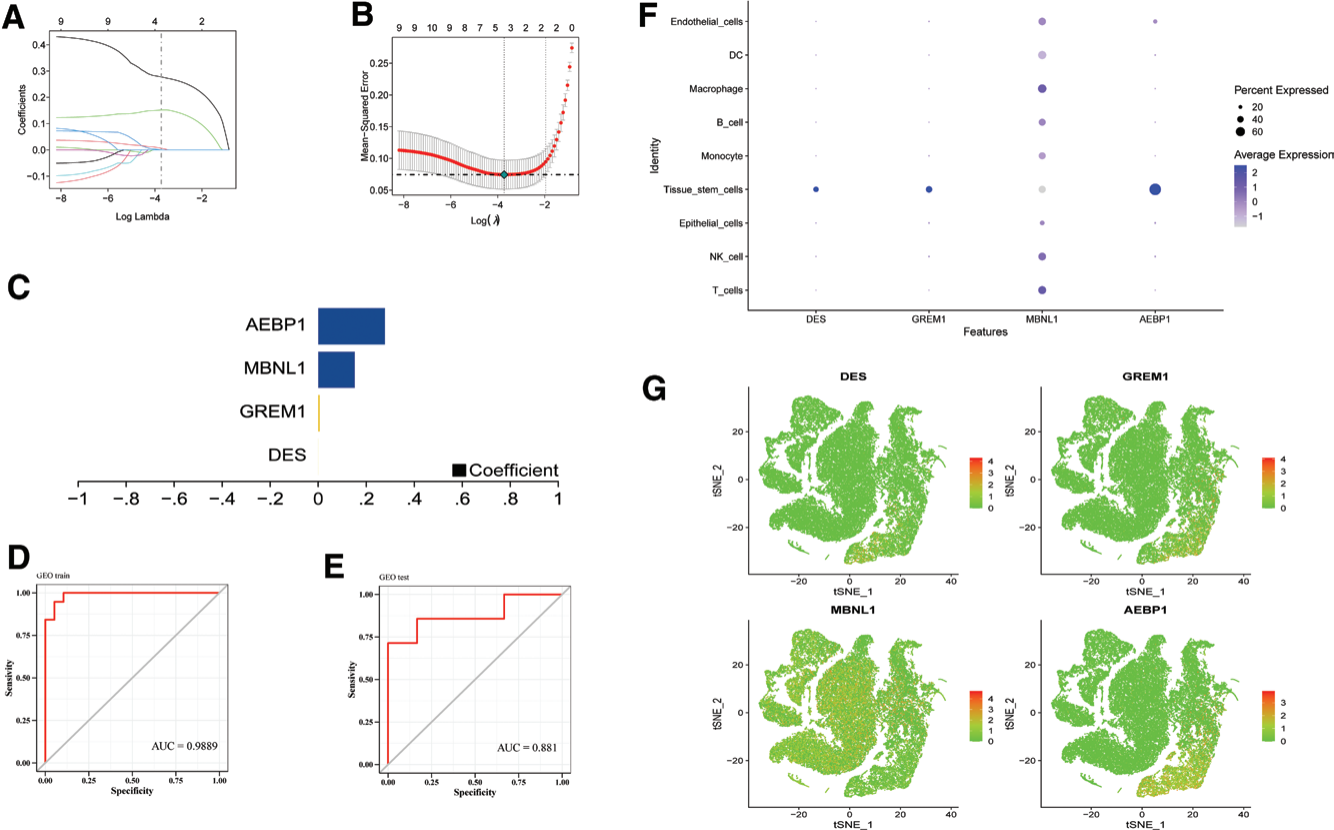
Construction of the diagnostic model. (A) The distribution of least absolute shrinkage and selection operator (LASSO) coefficients for differentially expressed genes. (B) Ten-fold cross-validation for the selection of tuning parameters in the LASSO model. (C) Histogram of LASSO coefficients for 7 key genes. (D, E) Prediction performance of the training set and validation set. (F, G) Scatter plot of the expression of key genes in single cells.

The GSE11691 and GSE7305 datasets served as the training set, whereas GSE25628 was used as the verification set. Feature selection through Lasso regression identified 10 intersecting differential genes. These 4 characteristic genes were identified and used as key genes to construct a prediction model for subsequent research (Fig. 4A and C). The model formula is: RiskScore = DES × 0.000117354399630161 + GREM1 × 0.00622875067401555 + MBNL1 × 0.151929696438042 + AEBP1 × 0.27771730829568. Evaluation of the diagnostic performance of the model revealed an impressive area under the curve (AUC) of 0.9889 (Fig. 4D). To further validate the model, we employed GSE25628 as an external dataset, confirming its strong stability with an area under the curve of 0.881 (Fig. 4E). We also examined the expression patterns of key genes in various single-cell types, including T cells, NK cells, epithelial cells, tissue stem cells, monocytes, B cells, macrophages, DC, and endothelial cells (Fig. 4F and G).

### 3.5. Analysis of immune infiltration of key genes

**Figure 5.**
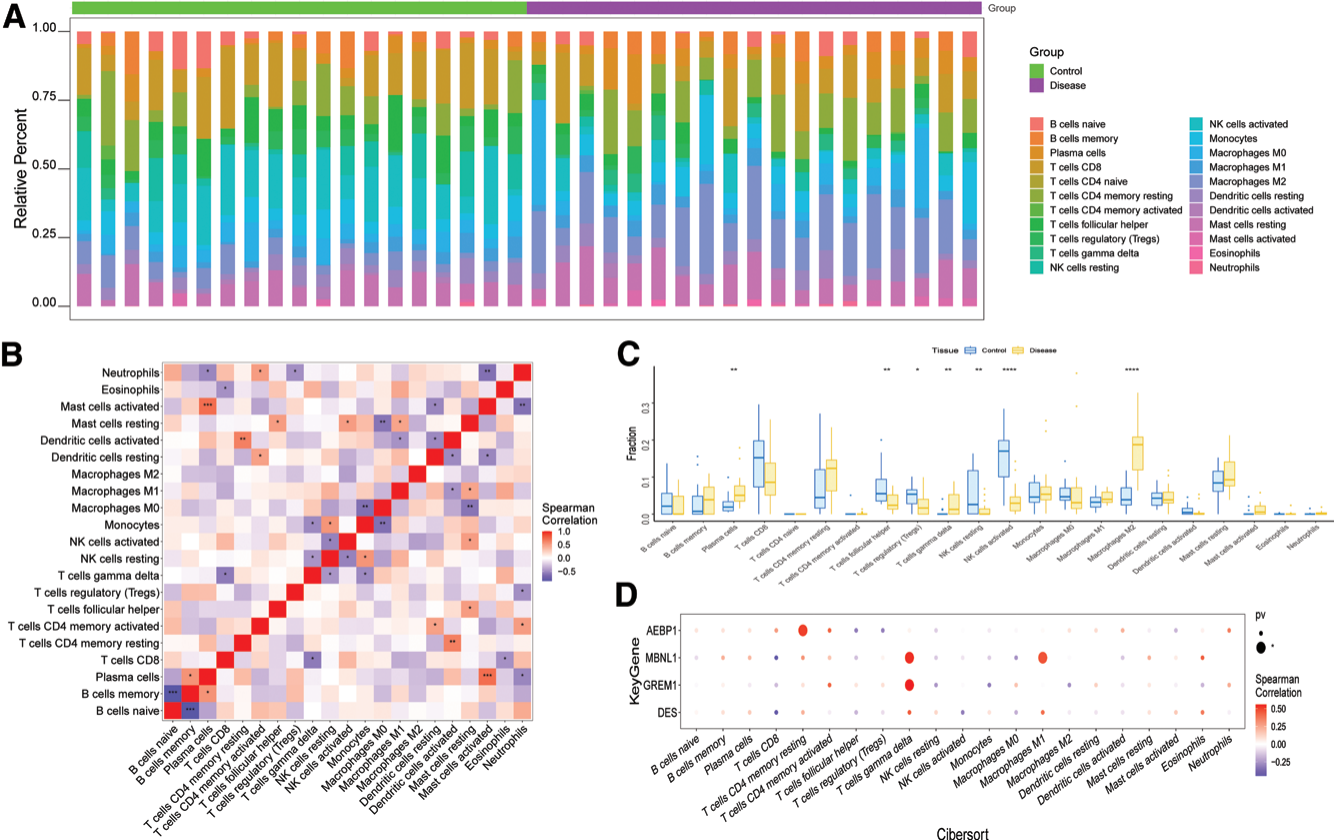
Immune infiltration in endometriosis. (A) Relative percentages of 22 immune cell subtypes. (B) Pearson correlation among the 22 immune cell types, with blue indicating positive correlation and red indicating negative correlation. (C) Differences in immune cell content between control samples and disease samples. (D) Pearson correlation between key genes and immune cells, with red indicating positive correlation and blue indicating negative correlation.

**Figure 6.**
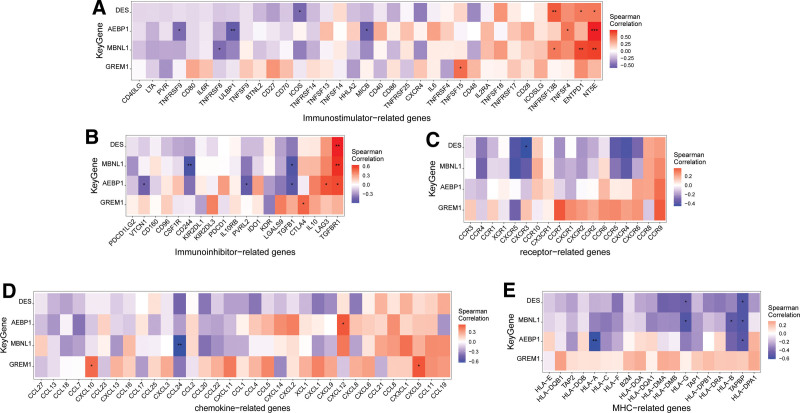
Relationship between key genes and immune factors. Pearson correlation heat map of key genes and immune factors, A-E represent immunostimulators, immunoinhibitors, receptors, chemokines, and MHC, respectively.

The microenvironment comprises fibroblasts, immune cells, extracellular matrix, growth factors, inflammatory factors, and unique physical and chemical characteristics. It significantly influences disease diagnosis, survival outcomes, and clinical treatment responses. The distribution of immune infiltration levels and the correlation heatmap of immune cell populations are shown in Figure 5A and B. Comparing the disease group with the normal group, there was a notable increase in plasma cells, gamma delta T cells, and Macrophages M2 were observed (Fig. 5C). Further exploration revealed strong correlations between key genes and immune cells, highlighting their crucial roles in the immune microenvironment (Fig. 5D). Additionally, our analysis demonstrated a close connection between these key genes and various immune factors including immunosuppressive factors, immunostimulatory factors, chemokines, receptors, and MHC-related genes (Fig. 6). Overall, these findings reinforce the significance of the key genes involved in immune cell infiltration and their pivotal roles in the immune microenvironment.

### 3.6. Signaling pathways involved in key genes

**Figure 7.**
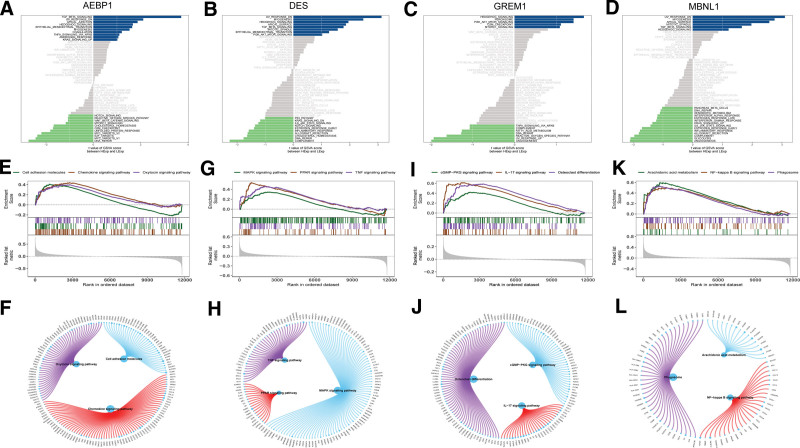
Pathways enriched for key genes. (A–D) Key genes from the high expression group and low expression group were scored for pathway activity using gene set variation analysis(GSVA), exploring the differences in pathways between high-risk group and low-risk group. The background gene set used was Hallmark. (E–L) The Kyoto Encyclopedia of Genes and Genomes (KEGG) signaling pathways associated with key genes, as well as the pathway regulation and the genes involved.

To further investigate the molecular mechanisms underlying the impact of the 4 key genes on endometriosis progression, we investigated the specific signaling pathways involved. GSVA revealed that Adipocyte Enhancer Binding Protein 1(AEBP1), characterized by high expression levels, was significantly enriched in the TGF_BETA_SIGNALING and UV_RESPONSE_DN pathways (Fig. 7A). Similarly, DES, with its elevated expression, showed enrichment in the PI3K_AKT_MTOR_SIGNALING and TGF_BETA_SIGNALING pathways (Fig. 7B). GREM1 was also highly expressed and enriched in the G2M_CHECKPOINT and MTORC1_SIGNALING pathways (Fig. 7C), while MBNL1 was enriched in the APICAL_SURFACE, HEDGEHOG_SIGNALING, and other signaling pathways (Fig. 7D). Moreover, GSEA results indicated that AEBP1 overexpression was associated with enrichment of cell adhesion molecules, chemokine signaling pathways, and the oxytocin signaling pathway (Fig. 7E and F). Similarly, high DES expression was enriched in the MAPK, PPAR, and TNF signaling pathways (Fig. 7G and H). Additionally, GREM1 overexpression correlated with enrichment in the cGMP − PKG signaling pathway, IL − 17 signaling pathway, osteoclast differentiation, and other signaling pathways (Fig. 7I and J). Lastly, MBNL1 overexpression showed enrichment in arachidonic acid metabolism, NF − kappa B signaling pathway, phagosomes, and other signaling pathways (Fig. 7K and L). These findings suggest that key genes may influence endometriosis progression through these pathways.

### 3.7. Analysis of transcriptional regulation related to key genes

**Figure 8.**
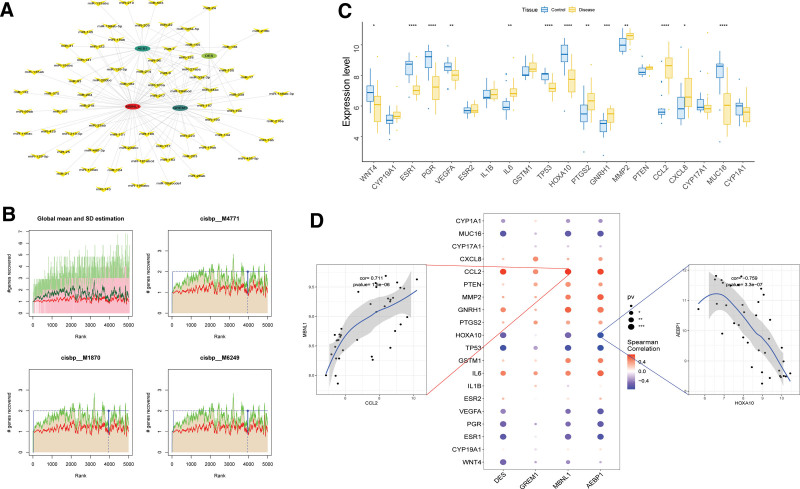
Motif transcriptional regulation analysis. (A) miRNA network of key genes, where large dots represent mRNA and small dots represent miRNAs. (B) The top 3 motifs had the highest AUC values. The red line represents the average recovery curve of each motif, the green line represents the average plus the standard deviation, and the blue line represents the recovery curve of the current motif. The maximum distance point (mean + SD) between the current motif and green curve indicates the selected maximum enrichment level. (C) Differential expression of disease-regulating genes in endometriosis, with blue representing control patients and yellow representing disease patients. (D) Correlation analysis between disease-regulating genes and key genes in endometriosis, with blue indicating a negative correlation and red indicating a positive correlation.

Furthermore, employing the miRcode database, we conducted reverse prediction for the 4 key genes, yielding an impressive set of 73 miRNAs and 140 mRNA-miRNA relationship pairs. For visual clarity, these relationships were depicted using Cytoscape (Fig. 8A). Remarkably, by subjecting these 4 key genes to gene set analysis, we discovered that they are regulated by shared mechanisms involving multiple transcription factors. Enrichment analysis of these transcription factors was performed using cumulative recovery curves. Motif-TF annotation and selection analysis of important genes showed that the motif with the highest NES (3.79) was cisbp _ M4771. We identified all enriched motifs and corresponding transcription factors of the key genes (Fig. 8B).

### 3.8. Correlation between key genes and disease regulatory genes

Endometriosis-related disease genes were obtained from the GeneCards database (https://www.genecards.org/), thereby enriching our understanding of this condition. Intriguingly, through meticulous analysis of the differences in the expression of disease genes between distinct patient groups, we observed notable variations in the expression levels of WNT4, ESR1, PGR, VEGFA, IL6, TP53, HOXA10, PTGS2, GNRH1, MMP2, CCL2, CXCL8, and MUC16 (Fig. 8C). Furthermore, by examining the expression levels of the 4 key genes, along with the top 20 genes based on the relevance score, we revealed significant correlations between the expression levels of key genes and multiple disease-related genes. In particular, MBNL1 exhibited a remarkably positive correlation with CCL2 (*R* = 0.711), whereas AEBP1 displayed a significant positive correlation with HOXA10 (r = −0.759) (Fig. 8D).

## 4. Discussion

Endometriosis is a complex biological process involving multiple genes and factors that results in a range of symptoms, varying degrees of severity, and diverse treatment responses.^[17]^ Hence, it is of paramount importance to investigate crucial genes associated with endometriosis. Using single-cell sequencing and transcriptome data, we successfully detected the key genes. A predictive model comprising 4 genes was constructed using LASSO regression and was subsequently verified for its diagnostic potential. Additionally, we examined the relationship between these 4 key genes and the immune status of endometriosis, identified pivotal pathways that may be implicated, and explored their correlation with the established regulatory genes associated with endometriosis. This comprehensive exploration will guide precise diagnosis and treatment strategies for endometriosis.

Endometriosis continues to pose challenges in terms of its underlying pathogenesis. Nonetheless, Dr John Sampson theory, which originated in the 1920s, has emerged as one of the most widely acknowledged explanations. According to his postulation, the primary causative factor of endometriosis is retrograde transport of menstrual debris.^[18]^ Endometrial stem/progenitor cells actively contribute to the development and progression of endometriosis by infiltrating the pelvic cavity during the retrograde menstruation. Under certain conditions, these cells undergo overactivation, leading to clonal expansion and the subsequent formation of new glands and stromal tissue.^[19,20]^ Menstrual endometrial stem cells display a greater propensity for self-renewal when compared to their proliferative or secretory counterparts,^[21]^ implying their potential as the primary cellular culprits behind lesion formation. Remarkably, our results corroborated these observations. Using single-cell sequencing of menstrual blood samples collected from individuals diagnosed with endometriosis, we found a strong correlation between endometrial stem cells and the pathogenesis of this condition. A recent study provided compelling evidence suggesting a potential link between early onset endometriosis and retrograde expulsion of endometrial stem cells into the pelvic cavity during neonatal uterine bleeding.^[22]^ Furthermore, early onset endometriosis tends to manifest with heightened severity compared to the adult form of the disease, frequently necessitating its classification as stage III or IV.^[23]^ These findings also suggest that endometrial stem cells may play a crucial role among the factors responsible for the onset of endometriosis. Consequently, the identification of marker genes associated with endometrial stem cells was chosen as the focus of subsequent investigations in this study.

The backflow of menstrual fragments elicits innate and adaptive immune reactions, prompting the infiltration of immune cells that seek to restore the peritoneal integrity. Subsequently, cytokines secreted by immune cells significantly contribute to the perpetuation of chronic inflammation, thereby supporting the growth and establishment of endometriotic lesions. In the early stages of endometriosis, a proinflammatory environment prevails; however, as the disease progresses, there is a shift towards immune tolerance.^[24,25]^ The presence of macrophages leads to oxidative stress, giving rise to chronic inflammation.^[26]^ In the context of endometriotic lesions, macrophages play a pivotal role in driving angiogenesis^[27]^ and facilitating fibrogenesis in endometriotic lesions.^[28]^ Impaired functionality of NK cells in endometriosis significantly contributes to immune evasion by ectopic endometrial fragments thriving within the peritoneal cavity.^[29]^ Amidst the focus of endometriosis, an extensive surge in plasma cells is observed alongside the crucial interplay between BLyS-responsive plasma cells and retrograde menstrual tissues, enabling the development of endometriosis lesions.^[30]^ In line with previous investigations, our study demonstrated a marked increase in macrophages and plasma cells, coupled with a significant reduction in NK cells within endometriosis foci. Moreover, we identified correlations between various immune cell populations present in the endometrial lesions. Although the verification of these relationships through additional experiments is pending, our results offer valuable insights and lay the groundwork for further analytical exploration in this particular area of research.

Our model incorporates 4 genes: AEBP1, MBNL1, GREM1, and DES. In an array of biological processes, AEBP1 is a multifunctional protein that encompasses adipogenesis,^[31]^ cell differentiation,^[32]^ and cholesterol homeostasis in the macrophages.^[33]^ The expression of AEBP1 intensifies as fibrosis worsens in nonalcoholic steatohepatitis (NASH), and its regulation is modulated by glucose, palmitate, and miR-372-3p.^[34]^ Inhibition of AEBP1 is an effective strategy to counteract renal fibrosis both in vivo and in vitro by suppressing the β-catenin signaling pathway.^[35]^ By negatively regulating IκBα, AEBP1 orchestrates an increase in macrophage inflammatory responsiveness by upregulating NF-κB.^[36]^ In the absence of direct experimental evidence for endometriosis, our findings revealed a marked increase in AEBP1 expression, specifically in the focal areas of this condition. Additionally, we established a noteworthy connection between AEBP1 and immune-related genes. Notably, a strong positive correlation was observed between AEBP1 and CXCL12 expression levels. The expression of CXCL12 in endometriosis pathological tissues^[37]^ coupled with its increased presence in the systemic circulation of affected patients^[38]^ significantly contributes to the underlying pathology of endometriosis. Additionally, this study highlights a negative correlation between AEBP1 and TGFB1, and a positive correlation between AEBP1 and TGFBR1. Transforming growth factor-beta (TGF-β) primarily functions as an anti-inflammatory cytokine,^[39]^ exerting immunosuppressive effects such as the inhibition or reversal of macrophage activation, leading to the downregulation of proinflammatory cytokine release.^[40]^ Reverse transcription-polymerase chain reaction (RT-PCR) analysis demonstrated significant downregulation of the anti-inflammatory cytokine TGF-β in endometriotic mesenchymal stem cells (MSCs).^[41]^ Suppression of TGFBR1 expression effectively impeded the viability and migratory capabilities of endometrial cells.^[42]^ Based on these studies, it has been postulated that AEBP1 plays a role in the pathogenesis and progression of endometriosis by modulating immune pathways. The upregulation of GREM1, a key player in early embryonic development and cyclical angiogenesis of the endometrium, was observed in both the eutopic endometrium and peripheral serum of patients diagnosed with endometriosis. These findings strongly suggest that GREM1 is involved in endometriosis pathogenesis.^[43]^ Our research results revealed a significant correlation between GREM1 and CXCL5 expression levels. Compared with endometriosis patients without deep infiltrating endometriosis (DIE), a significant elevation in CXCL5 levels was observed in the peritoneal fluid (PF) of patients with DIE.^[44,45]^ Additionally, our findings indicate a substantial positive correlation between GREM1 and CTLA4. The expression of CTLA-4 plays a crucial role in sustaining chronic inflammation observed in endometriosis, thereby influencing the development of infertility.^[46]^ Although DES and MBNL1 have not been extensively studied in the context of endometriosis, our investigation examined their correlation with pivotal endometriosis-related and immune microenvironment regulatory genes. Involvement of the HOXA10 transcription factor in the development of endometriosis is important.^[47]^ Through activation of the Akt and MAPK/Erk1/2 signaling pathways, the chemokine CCL2 exerts an autocrine effect to enhance the survival and invasiveness of endometrial stromal cells.^[48]^ The primary function of TP53 is to regulate the cell cycle progression and apoptosis. Aberrant TP53 expression has also been observed in endometriosis patients.^[49]^ Additionally, there is a strong association between TP53 polymorphisms and susceptibility to endometriosis.^[50]^ In this model, the 4 genes displayed a positive correlation with CCL2 and a negative correlation with TP53. Additionally, HOXA10 negatively correlated with DES, MBNL1, and AEBP1. Concurrently, we investigated the influence of miRNAs, transcriptional regulatory factors, and pathways on the regulation of these 4 pivotal genes, which yielded encouraging findings. The exceptional diagnostic value of this model can be attributed to the significance of these 4 genes in endometriosis. Although the exact mechanisms by which these genes contribute to endometriosis remain elusive, our analysis paves the way for further investigation, including the exploration of the immune microenvironment, understanding their interplay with known key genes, and uncovering regulatory pathways.

Our study has significant clinical implications because the diagnostic model has high diagnostic value. Furthermore, the 4 genes encompassed in the model exert crucial biological functions in endometriosis and have the potential to serve as therapeutic targets for endometriosis. However, it is essential to acknowledge the limitations of the present study. First, the retrospective design necessitates larger clinical cohort studies to comprehensively evaluate the effectiveness of the diagnostic models. Secondly, further validation of the mechanism involving the 4 core genes in endometriosis is warranted through in vivo and in vitro experiments. Finally, the limited number of subjects in this study emphasizes the need for multicenter and large-scale investigations to validate the effectiveness of the diagnostic model.

## 5. Conclusion

By employing scRNA-seq, WGCNA, and LASSO diagnostic model development methodologies, DES, GREM1, MBNL1, and AEBP1 were identified as pivotal core genes in endometriosis that display notable associations with tissue stem cell marker genes. These genes hold potential as diagnostic markers and molecular targets for therapeutic interventions in endometriosis.

## Acknowledgments

The authors thank the GEO website for sharing the data.

## Author contributions

**Conceptualization:** Yulin Song, Yu Zhang.

**Data curation:** Yulin Song, Le Wang.

**Formal analysis:** Yulin Song, Le Wang, Yu Zhang.

**Supervision:** Yu Zhang.

**Writing – review & editing:** Yu Zhang.

**Writing – original draft:** Yulin Song, Le Wang, Yu Zhang.

## Supplementary Material


