# Supplementary figures and images for: Identification of central genes for endometriosis through integration of single-cell RNA sequencing and bulk RNA sequencing analysis
Source: Medicine (Baltimore). 2023 Dec 15;102(50):e36707. doi: 10.1097/MD.0000000000036707 (PMC10727599; doi:10.1097/MD.0000000000036707)

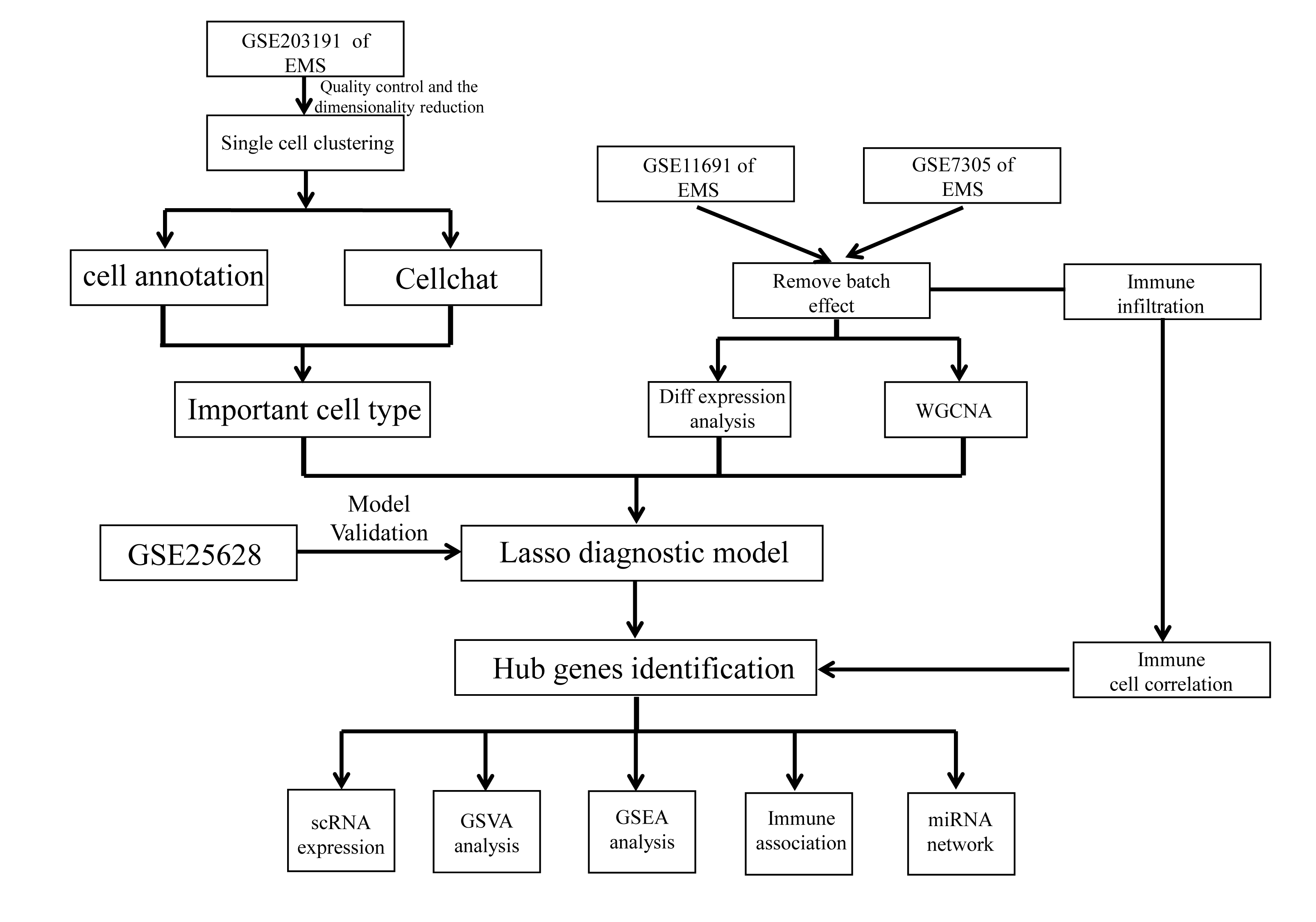

Supplement: Supplementary file 1 [file medi-102-e36707-s001.tif]
